# Retraction Note: Key wound healing genes as diagnostic biomarkers and therapeutic targets in uterine corpus endometrial carcinoma: an integrated in silico and in vitro study

**DOI:** 10.1186/s41065-026-00755-x

**Published:** 2026-10-10

**Authors:** Fuchuan Jiang, Sajjad Ahmad, Sadia Kanwal, Yasir Hameed, Qian Tang

**Affiliations:** 1 Department of Gynaecology and Obstetrics, Sichuan Provincial People’s Hospital, University of Electronic Science and Technology of China, Chengdu, Sichuan 610072 China; 2 Gomal Medical College, D. I. Khan, KPK Pakistan; 3 Al Nafees Medical College and Hospital Islamabad, Islamabad, Pakistan; 4 Department of Biochemistry, The Islamia University of Bahawalpur, Bahawalpur, Pakistan

**Retraction Note: Hereditas 162:5 (2025)**

**https://doi.org/10.1186/s41065-025-00369-9**

The Editor-in-Chief has retracted this article. After publication, concerns were raised regarding the in vitro images presented in Fig. 11 of this article, where the patterns of cells and colonies do not appear consistent with natural cell growth and proliferation. Additionally, the uncut western blot gel presented in the Supplementary Material appears to show contradictory results, with two proteins probed in adjacent lanes with different results. It has also been noted that a number of references in this article do not appear to support the statements preceding the citations.

The Editor-in-Chief therefore no longer has confidence in the presented data.

The authors have not responded to correspondence regarding this retraction.

